# Biogeographic Differences in the Microbiome and Pathobiome of the Coral *Cladocora caespitosa* in the Western Mediterranean Sea

**DOI:** 10.3389/fmicb.2018.00022

**Published:** 2018-01-23

**Authors:** Esther Rubio-Portillo, Diego K. Kersting, Cristina Linares, Alfonso A. Ramos-Esplá, Josefa Antón

**Affiliations:** ^1^Department of Physiology, Genetics and Microbiology, University of Alicante, Alicante, Spain; ^2^Working Group on Geobiology and Anthropocene Research, Institute of Geological Sciences, Freie Universität Berlin, Berlin, Germany; ^3^Departament de Biologia Evolutiva, Ecologia i Ciències Ambientals, Institut de Recerca de la Biodiversitat (IRBio), Universitat de Barcelona, Barcelona, Spain; ^4^Marine Research Centre of Santa Pola, University of Alicante, Alicante, Spain

**Keywords:** *Cladocora caespitosa*, necrosis, microbiome, pathobiome, Mediterranean Sea, amplicon sequencing, microbial diversity

## Abstract

The endemic Mediterranean zooxanthellate scleractinian reef-builder *Cladocora caespitosa* is among the organisms most affected by warming-related mass mortality events in the Mediterranean Sea. Corals are known to contain a diverse microbiota that plays a key role in their physiology and health. Here we report the first study that examines the microbiome and pathobiome associated with *C. caespitosa* in three different Mediterranean locations (i.e., Genova, Columbretes Islands, and Tabarca Island). The microbial communities associated with this species showed biogeographical differences, but shared a common core microbiome that probably plays a key role in the coral holobiont. The putatively pathogenic microbial assemblage (i.e., pathobiome) of *C. caespitosa* also seemed to depend on geographic location and the human footprint. In locations near the coast and with higher human influence, the pathobiome was entirely constituted by *Vibrio* species, including the well-known coral pathogens *Vibrio coralliilyticus* and *V. mediterranei*. However, in the Columbretes Islands, located off the coast and the most pristine of the analyzed locations, no changes among microbial communities associated to healthy and necrosed samples were detected. Hence, our results provide new insights into the microbiome of the temperate corals and its role in coral health status, highlighting its dependence on the local environmental conditions and the human footprint.

## Introduction

Mass mortality events of benthic invertebrates from different phyla (sponges, cnidarians, molluscs, ascidians, and bryozoans) have increased in frequency in the last two decades in the temperate Mediterranean Sea, with catastrophic effects in benthic communities (Cerrano et al., 2000; Linares et al., 2005; Garrabou et al., 2009; Crisci et al., 2011; Kersting et al., 2013; Kružić and Popijač, 2015; Jiménez et al., 2016; Rubio-Portillo et al., 2016a). Although the direct causes of these events remain unknown, there is scientific evidence to confirm that sea surface temperature anomalies linked to global warming are among the primary triggering factors (Garrabou et al., 2009; Kersting et al., 2013; Rivetti et al., 2014), together with energetic constraints (Coma et al., 2009) and the potential occurrence of thermodependent pathogens (Bally and Garrabou, 2007; Vezzulli et al., 2010; Rubio-Portillo et al., 2014).

*Cladocora caespitosa* is the only endemic zooxanthellate scleractinian reef-building coral in the Mediterranean Sea and is one of the invertebrates repeatedly affected by mass mortalities (Rodolfo-Metalpa et al., 2005; Garrabou et al., 2009; Kersting et al., 2013; Rubio-Portillo et al., 2016a). The occurrence of the recurrent mortality events in *C. caespitosa* has shown a significant association to positive thermal anomalies (Kersting et al., 2013) and the events were characterized by partial or total colony death due to polyp tissue necrosis (Rodolfo-Metalpa et al., 2005; Kersting et al., 2013). Extensive bioconstructions of this coral are very rare at the present time (Peirano et al., 1998) and a significant effort has been made to assess its response to different global change-related impacts, such as the increase of sea water temperature (Rodolfo-Metalpa et al., 2006a,b; Kersting et al., 2013). However, to better understand the response of this temperate coral to environmental changes it should be also taken into account that scleractinian corals form a great collaborative consortium with a wide range of different microbial partners (Rohwer et al., 2002), which play key functional roles and contribute to coral survival (Ritchie, 2006; Glasl et al., 2016) and heat tolerance (Ziegler et al., 2017). Indeed, it has been recently shown that the coral microbiome is one of the most complex microbial habitats studied to date (Blackall et al., 2015). The term microbiome describes the assemblage of microorganisms, active or inactive, associated with a habitat (Lederberg and McCray, 2001) and the core microbiome is comprised of the organisms that are common across the microbiomes from different habitats and likely play a key role within the habitat (Turnbaugh et al., 2007). Conversely, the term pathobiome is used to describe the consortium of microbes within the microbiome that play a direct role in the causation of disease (Vayssier-Taussat et al., 2014).

Meron et al. (2012) is the only work focused on examining the response of *C. caespitosa* associated microbial communities to environmental changes, in particular to decreased pH conditions by clone libraries. Here we provide the first deep sequencing taxonomic characterization of the *C. caespitosa* microbiome and its relation with geographic location and coral health status. As an initial step in understanding microbial community variability in *C. caespitosa* and its potential association to the occurrence of coral tissue necrosis, we have also identified the *C. caespitosa* core microbiome and pathobiome.

## Materials and Methods

### Sample Collection

**FIGURE 1:**
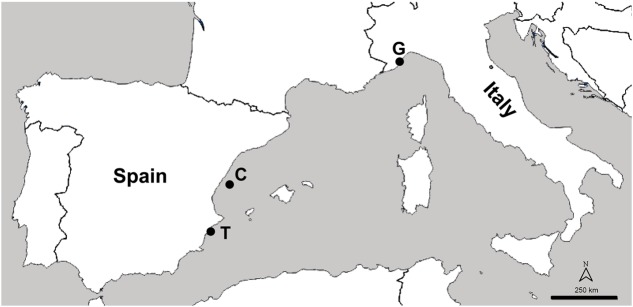
Map of the samplings location: G (Genova), C (Columbretes Islands), and T (Tabarca).

Thirty *C. caespitosa* samples were collected between 5 and 15 m depth in three different locations in the Western Mediterranean Sea: (1) Pietra Ligure (44°08′50.17″N, 08°17′04.02″E, Italy), a location on the Genova coast with an estimated human population of around 9,000 habitants; (2) Columbretes Islands (39°53′49.5″N, 00°41′12.8″E, Spain), a Marine Protected Area without permanent human population and situated at 30 nautical miles off the coast; and (3) Tabarca Island (38°09′59″N, 00°28′56″E, Spain) a Marine Protected Area 2 nautical miles off the coast which has a permanent population of 50–60 habitants, but with a highly seasonal tourism activity with more than 3,000 persons visiting the island each day in the summer (**Figure 1**). A fragment of six different colonies, three of them visually healthy and three with necrosis signs, were collected at Genova and Tabarca in 2012, at Columbretes in 2014 and at Tabarca and Columbretes in 2015.

### DNA Extraction and Polymerase Chain Reaction Amplification of 16S rRNA Genes

DNA was extracted from coral tissues using the UltraClean Soil DNA Kit (Mo Bio; Carlsbad, CA, United States) following the manufacturer’s instructions for maximum yield. The extracted genomic DNA was used for PCR amplifications of V3–V4 region of the 16S rRNA gene by using the following universal primers: Pro341F (CCTACGGGNBGCASCAG) (Takahashi et al., 2014) and Bact805R (GACTACHVGGGTATCTAATCC) (Herlemann et al., 2011). Each PCR mixture contained 5 μl of 10x PCR reaction buffer (Invitrogen), 1.5 μl of 50 mM MgCl2, 1 μl 10 mM dNTP mixture, 1 μl of 100 μM of each primer, 1 units of Taq polymerase, 3 μl of BSA (New England BioLabs), sterile MilliQ water up to 50 μl and 10 ng of DNA. Negative controls (with no template DNA) were included to assess potential contamination of reagents. The amplification products were purified with the GeneJET PCR purification kit (Fermentas, EU), quantified using the Qubit Kit (Invitrogen), and the quality (integrity and presence of a unique band) was confirmed by 1% agarose gel electrophoresis.

### Illumina High-Throughput 16S rRNA Gene Sequencing and Bioinformatic Analyses

The QIIME 1.8.0 pipeline (Caporaso et al., 2010) was used for data processing. Paired-end MiSeq sequencing of the 30 samples generated 2,481,629 reads, which were deposited in the NCBI Sequence Read Archive (SRA) database under BioProject PRJNA407809. Forward and reverse reads were merged using SeqPrep and classified to their respective samples according to their barcodes and then sequences were screened by quality and size, and de-replicated with the split_libraries.py script. The resulting file was checked for chimeric sequences with identify_chimeric_seqs.py script, against the SILVA_123 database^1^ (Quast et al., 2013) using UCHIME (Edgar et al., 2011). Operational taxonomic units (OTUs) were defined at the level of 99% similarity, close to the threshold used to distinguish species (98.7% similarity in the whole 16S rRNA gene, Stackebrandt and Ebers, 2006), followed by taxonomy assignments against the SILVA reference database (version 123) using the UCLUST algorithm (Edgar, 2010), using pick_open_reference_otus.py script. Singletons, OTUs with less than 0.05% of abundance, and OTUs classified as chloroplast or mitochondria were removed from the dataset. Due to the great difference in library size among samples, the OTU table was rarefied to 1,066 reads with single_rarefaction.py script (the lowest number of the post-assembly and filtered sequences in a sample) for comparisons across samples (Weiss et al., 2015). A non-rarefied dataset was also analyzed to confirm the sensitivity of our results after rarefaction and elimination of a portion of available data (data not shown). Alpha diversity metrics (total observed number of OTUs, and Shannon-Wiener diversity) were generated from the rarefied OTU table using alpha_diversity.py script. The similarity among different microbial communities was assessed using phylogenetic information using jackknifed UPGMA (weighted pair group method with arithmetic mean) clustering based on the weighted UniFrac (Lozupone and Knight, 2005) distances between samples implemented in the QIIME pipeline with jackknifed_beta_diversity.py script. The statistical significance of the cluster among samples was tested using PERMANOVA analysis and differences in diversity indexes using ANOVA analysis. All statistical analyses were performed in R with the ‘vegan’ package (Oksanen, 2011).

The core microbiome and pathobiome were analyzed using QIIME with the minimum fraction of samples set at 85 and 100%, lowest percentage at which core OTU abundance was stable across healthy and necrosed samples, respectively. For functional prediction, PICRUSt software package^2^ (Langille et al., 2013) was applied, which predicts the gene content of a microbial community from the information inferred from 16S RNA genes using an existing database of microbial genomes which predicts the tentative function of microbial communities. Metabolic predictions were made based on copy-number normalized OTUs and using healthy and samples showing tissue necrosis separately.

## Results and Discussion

### Assessment of the Bacterial Diversity and Changes in Community Composition in *Cladocora caespitosa*

A dataset of 2,145,856 high-quality partial 16S rRNA gene sequences (length 447.6 ± 17.7) was generated after merging, quality trimming and chimera detection from paired-end Illumina reads. We clustered reads into 33,707 OTUs at 99% similarity. 445 OTUs had an overall relative abundance over 0.05%. The results related to the richness indexes, including number of observed OTUs and Shannon’s diversity indexes are summarized in **Table 1**. Diversity differences were detected among locations (ANOVA, *F* = 11.478 and *p* < 0.001), being significantly higher in Columbretes than in Tabarca and Genova.

**Table 1:** Alpha diversity analysis of bacterial community associated with *Cladocora caespitosa*.

Location	Year	Health status	Replicates	Postfiltering reads	N° OTUs	Shannon diversity
Genova	2012	Healthy	1	9215	23	2.97
			2	9624	28	2.58
			3	216439	23	3.15
		Necrosed	1	8647	30	3.39
			2	4126	17	2.73
			3	5765	16	2.81
Columbretes	2014	Healthy	1	28129	29	2.50
			2	34848	59	4.20
			3	64999	45	3.87
		Necrosed	1	9171	67	4.75
			2	101108	51	3.26
			3	13549	43	3.85
	2015	Healthy	1	19508	38	2.64
			2	31399	45	2.82
			3	21020	61	3.86
		Necrosed	1	64999	38	2.64
			2	86562	30	2.45
			3	45963	50	2.44
Tabarca	2012	Healthy	1	4060	42	3.10
			2	180521	29	1.09
			3	40661	62	3.97
		Necrosed	1	14046	32	2.74
			2	1253	35	2.70
			3	1066	57	3.92
	2015	Healthy	1	2607	55	3.56
			2	121073	43	3.78
			3	44250	68	3.76
		Necrosed	1	97135	65	4.14
			2	93380	77	5.11
			3	97876	75	4.73


*OTUS and Shannon diversity index were calculated from normalized sequences with the lowest number of the post-assembly and filtered sequences in a sample (without singletons, OTUs with less than 0.05% of abundance and OTUs classified as chloroplast or mitochondria).*

PERMANOVA analysis (factors: location and health status) showed that the geographic location was the determining factor explaining differences in coral bacterial community composition (*F* = 0.4269, *p* < 0.001), but there was also a significant interaction between location and health status (*F* = 0.528, *p* < 0.001). In order to assess temporal variation in each location, we analyzed data from Tabarca and Columbretes, where samples were collected in two different years (factors: year and health status). While in Tabarca significant differences in community structure were found between years (*F* = 0.2729, *p* < 0.001) and coral health status (*F* = 0.1651, *p* < 0.01), in Columbretes only significant differences between years were found (*F* = 0.25503, *p* < 0.01). Therefore, the response or involvement of the microbial community in coral tissue necrosis seemed to be different in Columbretes than in Genova or Tabarca, where differences in microbial communities associated to coral health status were detected. These differences between geographic locations could be related to local environmental conditions, such as distance to the coast and human impact. It is well known that the diversity and abundance of *Vibrio* species, including coral pathogens, is higher in zones closer to the coast and with higher anthropic impact (Vezzulli et al., 2013; Rubio-Portillo et al., 2014), which, in turn, could contribute to geographic differences in coral susceptibility to bacterial infection.

### *Cladocora caespitosa* Bacterial Community Depends on Geographic Location

**FIGURE 2:**
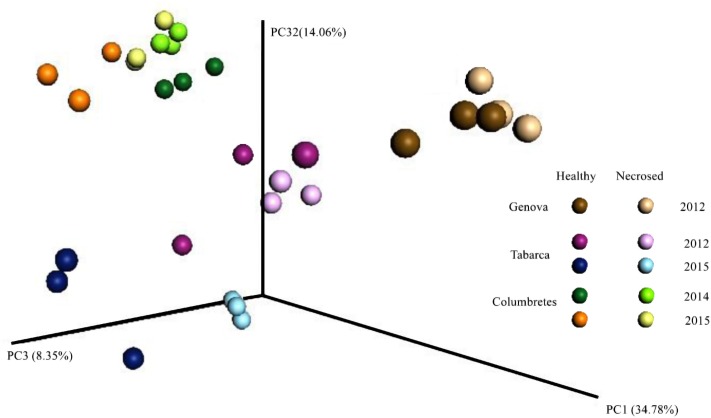
Bacterial communities associated with *Cladocora caespitosa* tissues clustered using coordinated analysis of the weighed UniFrac distance matrix. Each circle corresponds to a coral sample.

**FIGURE 3:**
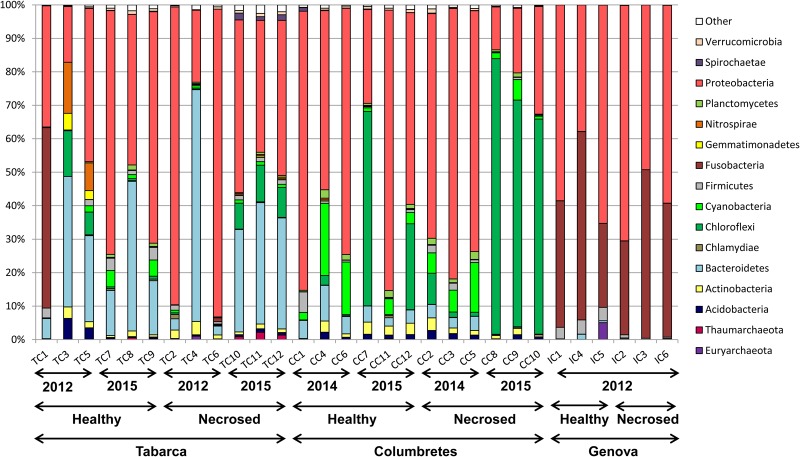
Taxonomic classification at the phylum level of bacterial communities associated with *C. caespitosa*.

Principal coordinate analysis using weighted UniFrac distances (Lozupone and Knight, 2005) clearly separated the samples by geographic location (**Figure 2**). Although Illumina 16S rRNA gene sequencing is not a suitable technique for absolute quantification purposes, as we have shown by analyzing mock communities (Rubio-Portillo et al., 2016b), it can be used to compare relative abundances among samples. The relative abundance at the phylum level (**Figure 3**) showed that the proportion of each phylum varied among corals from different locations. Differences in coral microbial communities depending on geographic location have been previously observed in *Acropora* species (Littman et al., 2009) and in *Seriatopora hystrix* (Pantos et al., 2015). Nonetheless, we found the phylum *Proteobacteria* (mainly Alpha and Gammaproteobacteria classes) to be ubiquitous and dominant in all *C. caespitosa* samples (20–90%), as previously found in other coral species, such as *Porites astreoides* (Wegley et al., 2007) and *Oculina patagonica* (Rubio-Portillo et al., 2016b). Coral samples also comprised other dominant phyla depending on location: *Fusobacteria* was more abundant in Genova (28–56%), *Chloroflexi* in Columbertes (1–64%) and *Bacteroidetes* in Tabarca (3–69%), although these last two phyla showed a great variability among samples within each location. The only study to date that has addressed microbiota associated with *C. caespitosa*, in Gulf of Naples, also found that *Proteobacteria* together with Bacteridetes were the dominant phyla in coral tissue (Meron et al., 2012), hence these phyla seem to be ubiquitous and dominant in *C. caespitosa* tissues regardless of geographic location.

Dissimilarity percentages calculated using SIMPER confirmed that the average dissimilarity among locations was high: 97.98% (Genova-Columbretes), 88.13% (Columbretes-Tabarca), and 84.20% (Genova-Tabarca). The OTUs primarily responsible for the biogeographical differences showed similarity percentages with known species below 97%, which could be indicative of the occurrence of new species. The largest representative sequences of each of these OTUs were inserted into the non-redundant SILVA SSURef_NR99_123 default tree (Quast et al., 2013) to identify the closest available sequences and then a phylogenetic reconstruction with the reference database LTP s123 (Munoz et al., 2011) was carried out using the ARB software package (Ludwig et al., 2004) (Supplementary Figure 1). In Genova the dominant sequence cluster was affiliated with *Propionigenium* lineage with 93.7–98.2% partial 16S rRNA gene sequence identity. Species of this genus are anaerobic bacteria that contribute to organic matter degradation in tidal sediments (Graue et al., 2012) and were previously associated with the gut environment of marine invertebrates (Dishaw et al., 2014). OTUs from Tabarca were loosely affiliated to *Maritimimonas* and *Tenacibaculum* genus with identity values of 93 and 89%, respectively. *Maritimimonas* genus is comprised of only one species *Maritimimonas rapanae*, which was isolated from gut microflora of a mollusk (Park et al., 2009) and *Tenacibaculum* genus has also been recently related to the gut microbiome of jelly fishes (Viver et al., 2017). Further studies are needed to assess if these taxa belong to the coral gut microbiota, but our results seem to indicate that corals living in different habitats with different nutritional resources could host different gut microbiota, which is consistent with previous studies in cold corals (Meistertzheim et al., 2016).

### Changes in the Bacterial Community Related to Coral Health Status

Bacterial community composition differed in relation to coral health status in Genova and Tabarca (**Figure 2**). In general terms, the microbial communities associated with colonies with tissue necrosis signs were more similar to each other than in healthy colonies in the two locations (**Table 2**, see similarity percentages). This fact has been previously observed in the coral *O. patagonica*, which also inhabits the Mediterranean Sea (Rubio-Portillo et al., 2016a). In addition to the OTUs belonging to *Propionigenium* and *Maritimimonas*, which could be part of the coral gut microbiome (see above), SIMPER analysis indicated that OTUs responsible for the observed differences (**Table 2** and Supplementary Figure 2) were *Thalassospira* and *Pseudovibrio* species, which were mainly associated with healthy corals in Genova and Tabarca, respectively. Species belonging to these two genera have been detected in microbial communities associated with other coral species and previous studies have emphasized their possible role in the coral holobiont. *Thalassospira* could be involved in carbon and phosphorus cycles (Thomas et al., 2010) and *Pseudovibrio* in the nitrogen cycle (Bondarev et al., 2013), as well as in the coral protection by inhibiting pathogens growth (Nissimov et al., 2009; Rypien et al., 2010). Nevertheless, *Vibrio* spp. were the OTUs mainly related to necrosed corals from both locations (**Tables 2A,B**), in agreement with previous findings that reported an increase of this bacteria genus in corals (Rubio-Portillo et al., 2014, 2016b) and gorgonians (Vezzulli et al., 2010) with disease signs.

**Table 2:** Percentage of contribution of main OTUs to bacterial community structure (based on SIMPER analysis), indicating the average contribution to the similarity (S) and dissimilarity (D) between healthy and unhealthy corals in each location: **(A)** Genova and **(B)** Tabarca.

(A) Genova		

OTU	Healthy	Unhealthy	Healthy/unhealthy
	*S* = 46.66	*S* = 60.79	*D* = 58.84
*Vibrio scophthalmi*	5.66	12.92	14.25
*Arcobacter* sp.	0.38	6.16	8.97
*Thalassospira* sp.	5.77	0.19	6.41
*Propionigenium* sp.1	44.31	28.49	5.33
*Propionigenium* sp. 2	17.83	12.77	5.26

**(B) Tabarca**		

**OTU**	**Healthy**	**Unhealthy**	**Healthy/unhealthy**
	***S* = 28.43**	***S* = 41.02**	***D* = 87.53**

*Pseudovibrio* sp.	17.06	1.82	28.64
*Vibrio alginolyticus*	0.17	9.80	11.72
*Vibrio* sp. *FPLN2*	0.22	8.37	10.54
*Maritimimonas* sp.	2.94	5.28	5.09


Conversely, with the level of resolution provided by 16S rRNA gene profiling, no significant differences were found in the microbial community between healthy and necrosed corals in Columbretes, which may suggest that bacteria are not directly involved in the development of tissue necrosis in this location. Furthermore, the coral pathogen *Vibrio coralliilyticus* was detected both in necrosed and healthy corals in Columbretes (see below), which adds additional uncertainty to the role that microorganisms might play in *C. caespitosa* necrosis in this location. It is well known that interactions among coral pathogens, environmental stresses and physiological and immune status of the coral host are complex (Bourne et al., 2009) and it would be necessary to carry out further studies to better understand the role of microorganisms in the necrosis events. Therefore, *Vibrio* species could be transient members that can vary in response to geographic location and other environmental factors.

### Temporal Changes in *Cladocora caespitosa* Bacterial Community

SIMPER analysis showed that in 2015, when an exceptional heat wave was recorded in Tabarca (Rubio-Portillo et al., 2016a) and in Columbretes (unpublished results), *C. caespitosa* bacterial community associated with corals with apparently different health status were more similar to each other than to those collected in previous years (**Table 3**, see similarity percentages) in the same locations. Therefore, coral microbial communities’ changes due to heat stress were detected in both healthy and necrosed corals. In Columbretes an increase in species belonging to *Chloroflexi* phylum was detected, while in Tabarca a decrease in *Pseudovibrio* genus and an increase of *Vibrio* and *Ruegeria* genus was detected (**Table 3**). Species belonging to *Vibrio* genus are well known coral pathogens but *Ruegeria* genus has also been previously linked to different coral diseases, such as Black Band Disease in the Caribbean Sea (Sekar et al., 2008), Yellow Band Disease in the Red Sea (Apprill et al., 2013) or White Patch Syndrome in the Indian Ocean (Séré et al., 2013), although it has never been demonstrated to be the etiological agent of these coral diseases. Therefore, in Tabarca heat stress seemed to be accompanied by an increase of potential coral pathogens in coral microbial communities. Similarly, the microbial community associated with this coral species also showed some changes in bacterial group composition under different pH conditions (Meron et al., 2012), so environmental stress related to climate change could influence *C. caespitosa* microbial community.

**Table 3:** Percentage of contribution of main OTUs to bacterial community structure (based on SIMPER analysis), indicating the average contribution to the similarity (S) and dissimilarity (D) between corals collected in different years in **(A)** Columbretes and **(B)** Tabarca.

(A) Columbretes		

OTU	2014	2015	2014/2015
	*S* = 12.5	*S* = 42.76	*D* = 90.30
Uncultured Chloroflexi bacterium	34.67	468	23.02
Uncultured *Cohaesibacter*	1.33	98.67	5.17
Uncultured *Rhodothermaceae*	89.93	0	4.67
Uncultured *Anaerolineaceae*	9.67	90.50	4.64

**(B) Tabarca**		

**OTU**	**2012**	**2015**	**2012/2015**
	***S* = 8.39**	***S* = 41.67**	***D* = 90.58**

*Ruegeria* sp.	15.83	153.5	8.36
*Pseudovibrio* sp.	142.5	57.83	8.22
*Vibrio* sp.	8.00	97.83	7.91
Uncultured *Blattabacteriaceae*	93.83	1.33	5.03


### The Core Microbiome of *Cladocora caespitosa*

**FIGURE 4:**
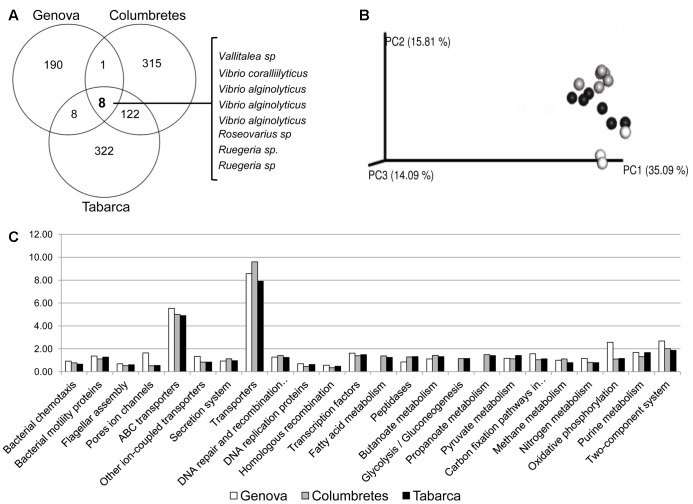
Analysis of the core microbiome associated with *C. caespitosa*. **(A)** Venn Diagrams showing OTUs shared by core microbiomes; **(B)** phylogenetic cluster of samples using coordinated analysis of the weighed UniFrac distance matrix, each circle corresponds to a coral sample (in white Genova, in gray Columbretes and in black Tabarca); and **(C)** most abundant predicted functions from the core microbiome using PICRUSt and copy-number normalized OTUs from healthy corals samples.

Using Venn diagrams (**Figure 4A**), we have identified a small group of bacteria belonging to the genera *Vallitaela*, *Vibrio*, *Roseovarius*, and *Ruegeria* that are ubiquitously associated with *C. caespitosa* regardless of local environmental factors, and could thus be considered as the coral core microbiome. These bacterial phylotypes, which had less than 4% relative abundance within the microbiome, are rare but conserved among geographic locations and time and they probably play key roles in the coral holobiont, as suggested by Ainsworth et al. (2015). Indeed, some of these OTUs like *Vibrio* spp. and *Roseovarius* sp., have been previously related to nitrogen (Chimetto et al., 2008) and sulfur cycling (Raina et al., 2009) in corals. Noticeably, the coral pathogen *V. coralliilyticus*, previously related to diseases in the red gorgonian *Paramuricea clavata* (Bally and Garrabou, 2007; Vezzulli et al., 2010) and the coral *O. patagonica* (Rubio-Portillo et al., 2014), forms part of the *C. caespitosa* core microbiome. This is in agreement with our recent results that showed that healthy and necrosed samples from *C. caespitosa* harbor different clonal types of *V. coralliilyticus*, which could, therefore, have different invasive-disease potential (Rubio-Portillo et al., 2017). This pathogen was also detected in apparently healthy tissue of the coral *Montastrea annularis* (Rypien et al., 2010), which is in good agreement with our findings. The role of the pathogen *V. coralliilyticus* in *C. caespitosa* microbiome is not clear and requires further research, but this species seems to be a native member of *C. caespitosa* coral microbial community, which in particular conditions (e.g., heat stress) could produce tissue necrosis.

Venn Diagrams only show differences among samples based on presence/absence of OTUs, but different OTUs that belong to the same lineage could perform similar functions within the coral holobiont (Shade and Handelsman, 2012). Therefore, UniFrac distances analysis was used to assess differences in phylogenetic diversity across samples. This analysis did not show differences in the core microbiome of samples collected at the three different locations (**Figure 4B**) and putative functions, predicted from 16S RNA gene information with PICRUSt, were also very similar (**Figure 4C**). Most of these functions were linked to energy metabolism, such as the nitrogen cycle or carbon fixation, or related to transport of sugars and ions, i.e., to metabolic exchange between the coral host and bacterial microbiota. Therefore, OTUs that constituted the core microbiome were different among locations but they were phylogenetically closed and they carry out similar functions in the coral holobiont.

### The Pathobiome of *Cladocora caespitosa*

**FIGURE 5:**
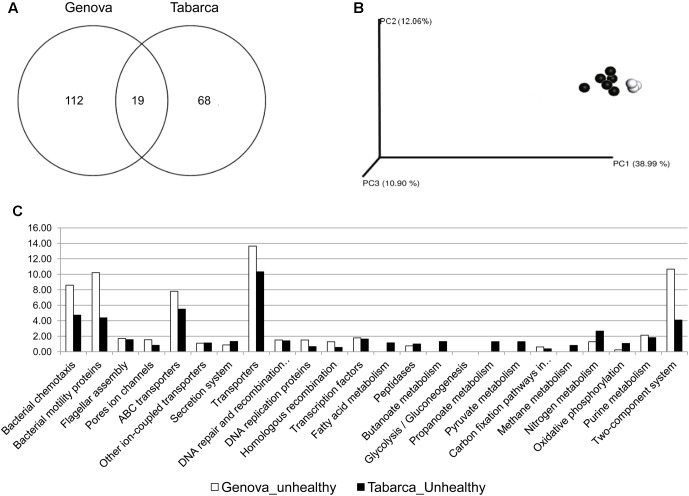
Analysis of pathobiome associated with *C. caespitosa*. **(A)** Venn Diagrams showing OTUs shared by pathobiomes; **(B)** phylogenetic cluster of samples using coordinated analysis of the weighed UniFrac distance matrix, each circle corresponds to a coral sample (in white Genovan and in black Tabarca); and **(C)** most abundant predicted functions from the pathobiome using PICRUSt and copy-number normalized OTUs from corals samples with tissue necrosis signs.

The *C. caespitosa* pathobiome was assessed only from necrosed samples collected in Tabarca and Genova, the two locations where the coral bacterial associated community was different among healthy and necrosed corals. Nineteen OTUs (**Figure 5A**) constitute the *C. caespitosa* pathobiome and 15 of them belong to *Vibrio* genus, including the two known coral pathogens *V. mediterranei* and *V. corallilyticus*. This is worth noting since a previous study suggested a synergistic effect on the virulence of these two pathogens (Rubio-Portillo et al., 2014). UniFrac distances analysis did not show differences in the core pathobiome of samples collected in these two locations (**Figure 5B**). Furthermore, virulence-associated factors, involved in motility, chemotaxis and two-component regulatory signal transductions systems, were the functions predicted for the species present in these pathobiomes (**Figure 5C**), which also suggested that *Vibrio* species could be related to the increase of virulence factors in corals with tissue necrosis signs, and could thus be responsible for this process in Genova and Tabarca.

## Conclusion

Recently, Hernandez-Agreda et al. (2017) suggested that the coral microbiome is divided into three main components: (i) a ubiquitous core microbiome; (ii) a dynamic site and/or species-specific community; and (iii) a highly variable community reflective of biotic and abiotic fluctuations. Here, we show that in *C. caespitosa* the ubiquitous core microbiome is constituted by rare conserved OTUs, including the coral pathogen *V. coralliilyticus*, which could be involved in nutrient cycling in the coral holobiont. The site-specific community is composed of OTUs that may be related to the gut microbiome and hence depends on nutritional resources and environmental conditions. The pathobiome of *C. caespitosa* was constituted entirely by *Vibrio* species, including the pathogens *V. coralliilyticus* and *V. mediterranei*. This fact, together with the increase of virulence factors predicted in the pathobiomes, suggests that these pathogens could be involved in *C. caespitosa* tissue necrosis in Tabarca and Genova, but not in Columbretes, the most pristine of the three analyzed locations. Accordingly, as previously hinted (Kersting et al., 2013, 2015), *C. caespitosa* tissue necrosis likely has a multi factorial origin, with the increase of sea water temperature as the main triggering factor (Kersting et al., 2013), while the role of the coral microbial community in the necrosis events could be highly dependent on local environmental conditions and the human footprint.

## Ethics Statement

The sampling in the Mediterranean Sea was performed in accordance with Spanish and Italian laws. In particular, for sampling in the Marine protected Areas of Tabarca and Columbretes permissions were granted by the Secretary-General for Fisheries of the Spanish Ministry of Agriculture, Food and Environment (permission numbers are 03/12 and 09/15 for Tabarca and 04/14 and 06/15 for Columbretes). For sampling in Pietra Ligure local competent authorities (Coast Guard and Municipality of Pietra Ligure) were informed and allowed the sampling of biological material for research purposes only. Italian laws do not ask for permissions for sampling in case of scientific purposes.

## Author Contributions

DKK and ER-P collected the samples. ER-P performed the DNA extractions, analysis of results. All authors contributed to the manuscript and participated in the writing and editing process.

## Conflict of Interest Statement

The authors declare that the research was conducted in the absence of any commercial or financial relationships that could be construed as a potential conflict of interest.

## Footnotes

1 http://www.arb-silva.de

2 http://picrust.github.com/picrust/
