# Supplementary figures and images for: Biogeographic Differences in the Microbiome and Pathobiome of the Coral Cladocora caespitosa in the Western Mediterranean Sea
Source: Front Microbiol. 2018 Jan 23;9:22. doi: 10.3389/fmicb.2018.00022 (PMC5787083; doi:10.3389/fmicb.2018.00022)

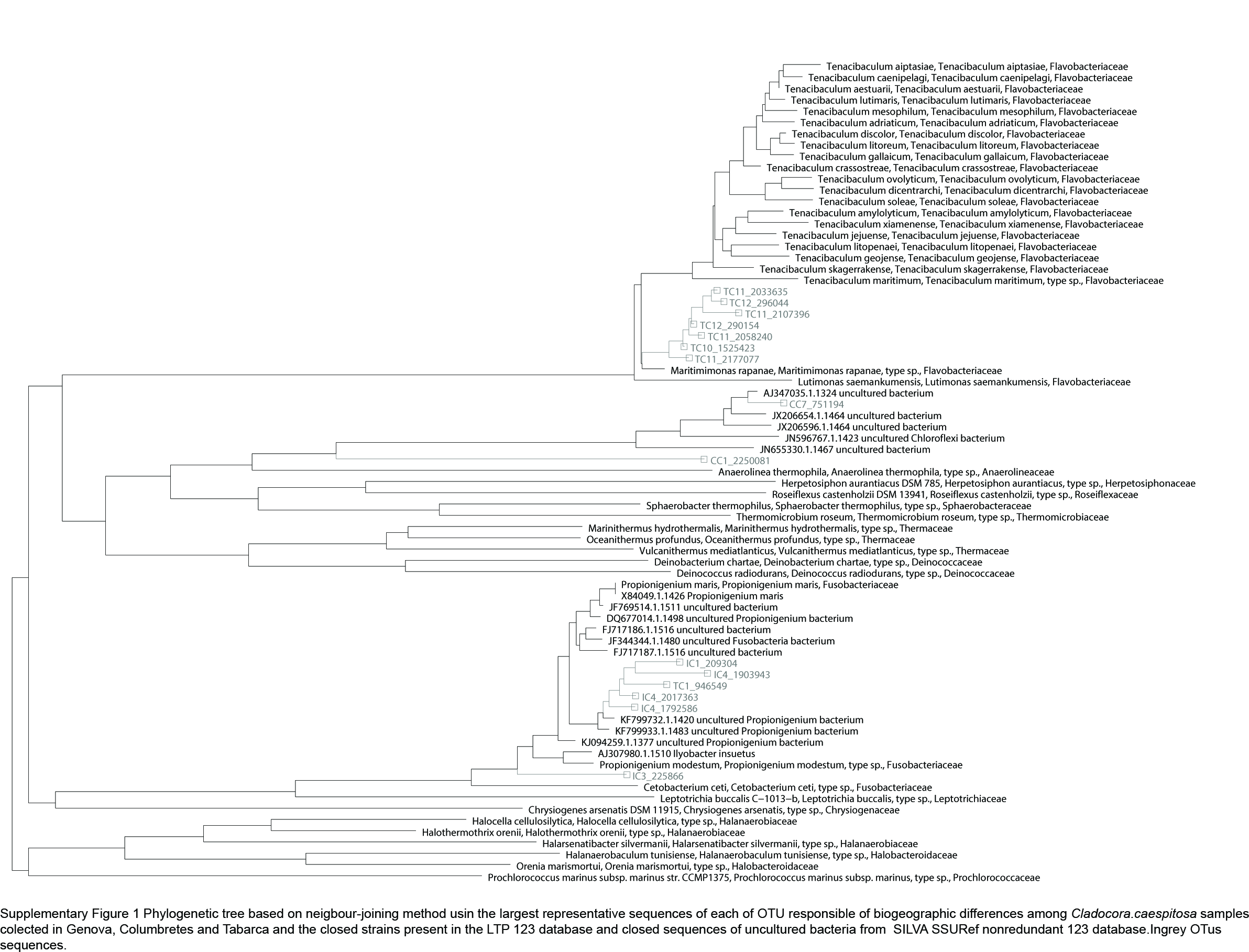

Supplement: Supplementary file 1 [file Image_1.TIF]

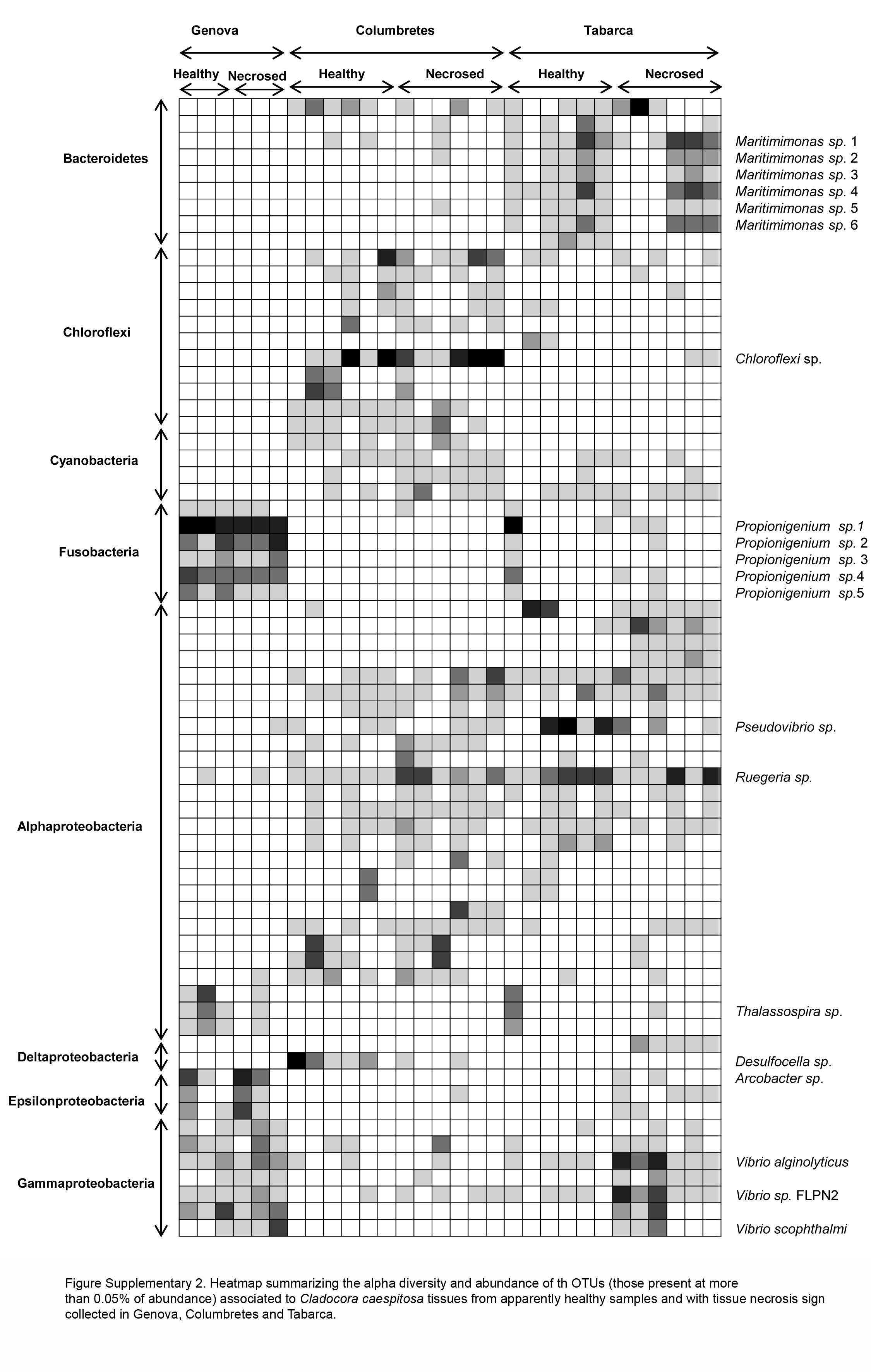

Supplement: Supplementary file 2 [file Image_2.tif]
